# The influence of threatening visual warnings on tobacco packaging: Measuring the impact of threat level, image size, and type of pack through psychophysiological and self-report methods

**DOI:** 10.1371/journal.pone.0184415

**Published:** 2017-09-14

**Authors:** Olivier Droulers, Karine Gallopel-Morvan, Sophie Lacoste-Badie, Mathieu Lajante

**Affiliations:** 1 IGR-IAE Graduate School of Management–CREM UMR 6211, University of Rennes 1, Rennes, France; 2 EHESP School of Public Health–EA 7348 MOS, Rennes, France; 3 IUT GEA–CREM UMR 6211, University of Rennes 1, Rennes, France; 4 Faculty of Business Administration—Marketing Department, Laval University, Quebec City, Québec; Boston Children's Hospital / Harvard Medical School, UNITED STATES

## Abstract

The first aim of this research was to assess the effectiveness, in terms of emotional and behavioral reactions, of moderately vs. highly TVWs (Threatening Visual Warnings) displayed on tobacco packs. Given the key role that emotional reactions play in explaining the effect of TVWs on behaviors, psychophysiological and self-report methods were used–for the first time in this context–to measure the emotions provoked by TVWs. The second aim of this research was to determine whether increasing the size of warnings, and their display on plain packaging (compared with branded packaging) would improve their effectiveness.

A within-subjects experiment was conducted. Three variables were manipulated: health warning threat level (high vs. moderate), image size (40% vs. 75%) and pack type (plain vs. branded). A convenience sample of 48 French daily smokers participated. They were exposed to eight different packs of cigarettes in a research lab at the University of Rennes. Smokers’ emotions and behavioral intentions were recorded through self-reports. Emotions were also evaluated using psychophysiological measurements: electrodermal activity and facial electromyography. The results revealed that TVWs with a high threat level are the most effective in increasing negative emotions (fear, disgust, valence, arousal) and behavioral intentions conducive to public health (desire to quit, etc.). They also highlight the appeal of increasing the size of the warnings and displaying them on plain packs, because this influences emotions, which is the first step toward behavioral change.

Increasing the threat level of TVWs from moderate to high seems beneficial for public health. Our results also confirm the relevance of recent governmental decisions to adopt plain packaging and larger TVWs (in the UK, France, Ireland, Canada, New Zealand, Hungary, etc.).

## Introduction

Tobacco use kills nearly six million people every year worldwide [1]. The WHO Framework Convention on Tobacco Control (FCTC) is a response to this pandemic, providing various support tools to develop effective tobacco control policies [2]. The present study focused on one of these tools, namely the TVWs (Threatening Visual Warnings) placed on cigarette packs and implemented in over 100 countries [3].

Some researchers have found that TVWs have little or no effect on people’s reactions and behavioral intentions with respect to smoking [4, 5, 6]. These findings may be attributable to the cognitive dissonance that smokers experience: they may resist TVWs by minimizing the threat, rejecting the problem, or avoiding the message [7, 8]. Conversely, other studies have shown TVWs to have a positive influence on smokers’ desires to quit or reduce tobacco consumption [9–15], and in dissuading people from starting to smoke [16–18]. In addition, most experimental studies have shown that TVWs are more effective than textual warnings in terms of increasing attention, credibility, and negatively affecting attitudes toward smoking and behavioral intentions [19]. According to some researchers, this effect can be explained by the central role of emotional reactions in the persuasion process [20–25]: the more threatening and aversive the TVWs, the stronger the negative emotions elicited (fear, disgust), and the more likely individuals will be motivated to quit or not to start smoking.

Due to these conflicting results, some researchers have concluded that “*the evidence for or against the use of pictorial warnings is insufficient*” [26, p.11]. Thus, further research is needed. Given the key role that emotional reactions play in explaining the effect of TVWs, this aspect requires greater understanding. Most studies on TVWs have used self-report methods to measure emotional reactions [27], which serve as the basis for the conscious representation of emotional processes [28, p.497]. However, these methods have limitations [7, 29, 30], such as neglecting the unconscious emotional reactions involved in different subsystems (physiological reactions). To reduce the limitations of measuring emotions with verbal measurements only, neuroimaging has been used in recent research [31–32]. Results have highlighted that highly TVWs were associated with greater activation in brain regions involved in cognitive/affective decision-making, memory and emotional reactions, which lead to stronger self-reported motivation to reduce or stop smoking. An electroencephalographic study also revealed that TVWs with strong (compared with weak) negative emotional content were more effective in reducing the brain and behavioral correlates of smoking addiction [33]. It is important to continue developing these non-verbal methods to better understand the emotional impact of TVWs, as affective reactions are the first step toward behavioral change. Therefore, the aim of our research was to analyze, by combining self-reports (questionnaires) and non-verbal measurements (psychophysiological ones), whether increasing the threat level of TVWs (moderate vs. high) enhances the emotional and behavioral reactions of smokers. We compared moderately and highly TVWs because many countries have now adopted these threat levels and most research, to date, has analyzed the effect of low (text-only warnings) vs. high threat levels (visual warnings) [19]. We also answered questions that policymakers are faced with today: would increasing the size of warnings and displaying them on plain (removal of all branding: logos, colors, etc.) rather than branded tobacco packs enhance their effectiveness? The few studies that have been conducted on the multiplier effect of the size of warnings and the context in which they are used (plain vs. branded packaging), have produced promising results: larger TVWs seem to increase the desire to quit, to reduce tobacco consumption and to seek help quitting [12, 34, 35]; larger TVWs placed on plain (vs. branded) packs seem to have no impact on cravings to smoke or on the desire to stop smoking [36]; and plain (vs. branded) packs significantly increase the impact of TVWs on cravings and evoked fear [37]. More research is needed to better understand the combined effect of these prevention tools.

## Method

To answer our research questions, an experiment using self-reports and psychophysiological measurements was carried out on smokers. It involved measuring electrodermal activity to evaluate the intensity of emotional reactions (arousal) and facial electromyographic activity to evaluate their polarity (emotional valence) [38, 39].

### Design and materials

Three variables were manipulated in this experiment: the threat level of TVWs (moderate vs. high), the image size (40% vs. 75%) and the type of packaging (branded vs. plain). TVWs similar to warnings used in various countries were selected from different sources (a warning currently used in European countries [40], a warning used in Chile in 2006 [41], and photographs available on the Internet, via Google images, at the time this research was conducted). Eight pictures were selected: four, presumably highly threatening, and four, presumably moderately threatening (S1 Appendix). They were categorized as “high”or “moderate”according to recommendations from previous research that used multiple threat levels [42]. For the highly threatening pictures, close-up photographs with vivid depictions of the physical effects of diseases were selected (e.g., a close-up of a tongue with cancer/a foot with gangrene). For moderately threatening photographs, wide-angle images with fewer visible physical effects of diseases were chosen (a man who had lost a leg due to gangrene sitting in a wheelchair/the face of a man with a hole in his throat due to cancer). A pretest on 165 people was conducted to validate this categorization. People were exposed to the eight pictures and were asked to answer two questions: “*When you look at this picture*, *do you feel the following emotions*: *I am afraid / I feel disgusted”*; this was accompanied by a five-point Likert scale (1: strongly disagree; 2: disagree; 3: moderately agree; 4: agree; 5: strongly agree). Of the 165 questionnaires completed, 163 were considered valid. The mean age of the sample was 18.32, 31.9% were female, and 34.4% were smokers. As expected, the mean scores of the four moderately threatening images were significantly lower than the mean scores of the four highly threatening images on both measures: “*I am afraid*” (M_moderate_ = 2.68, M_high_ = 3.42, t = -13.15; *p* < .0001) and “*I feel disgusted*” (M_moderate_ = 2.79, M_high_ = 4.60, t = -30.75; *p* < .0001).

The eight pictures selected were displayed on current, branded tobacco packs and on plain packs in two sizes with coverage of 40% (the size for visual warnings in Europe before 2017) and 75% (the current size in Canada). The plain packaging characteristics currently used in Australia (since 2012), in France, and in the United Kingdom (since 2016) were applied: dark green color (Pantone 448C), brand name in gray (Pantone Cool Gray) and Lucida Sans typeface. The eight pictures were combined with two textual health statements that corresponded to the disease they present: “Smoking clogs your arteries” and “Smoking causes mouth and throat cancer”. These two messages were chosen because they are currently used in the European Union [40] and in other countries (Australia, Chile, Brazil, etc.). Two different photographs that depicted each disease were selected for each of the threat levels (high/moderate). A professional designer put the eight photographs and the two text messages onto packs of three well-known cigarette brands in France: Marlboro, Camel, and Lucky Strike. The text message was written in white and displayed at the bottom of the pictures against a black background (a format currently used in various countries). In total, 96 different representations of packs were created (8 pictures x 2 image sizes x 2 types of packs x 3 brands).

### Self-reports and psychophysiological measurements

Two theoretical models were used to understand and identify individuals’ emotions: the categorical approach, which measures basic emotions such as fear, disgust, etc. [43, 44] and the dimensional approach, which measures the dimensions of pleasure (valence) and arousal [45]. Valence is defined as a displeasure-pleasure (negative-positive) continuum corresponding to the participant’s readiness to approach or withdraw from the stimulus. The arousal dimension is defined as a calm-excitement continuum which relates to the participant’s level of alertness or activation.

The self-reported negative emotions generated by the TVWs were evaluated by the verbal scale: “*When you look at this pack of cigarettes*, *do you feel the following emotions*? *I am afraid / I feel disgusted*” [46]. These two questions were accompanied by a five-point Likert scale (1: strongly disagree; 2: disagree; 3: moderately agree; 4: agree; 5: strongly agree). The average of the two items was calculated to represent the level of negative emotions felt (Cronbach’s alpha = 0.708). The self-report iconic SAM (Self-Assessment Manikin) scale was used to measure valence (pleasure/displeasure) and arousal in emotional reactions [47] (S2 Appendix).

Physiological arousal was evaluated by measuring electrodermal activity (EDA), an indicator of autonomic nervous system activation. To measure it, two electrodes were placed on the middle phalanges of the index and middle fingers of the non-dominant hand. The EDA signal was analyzed using LEDALAB V3.3.2 analysis software: phasic activity (i.e., the stimulus-related activity) was extracted from the tonic activity (i.e., the baseline) of the EDA by using a continuous decomposition analysis (CDA), and a threshold criterion of 0.01μSiemens was applied to the extracted phasic activity to determine the physiologically significant electrodermal responses. Usually, the amplitude of the phasic activity is between 0.1 and 2μSiemens. Finally, EDA scores represent the *Integrated Skin Conductance Responses* (ISCR) of the phasic component, recorded over a duration of 4,500ms, beginning at the onset of the stimulus [48–50].

Physiological valence was measured through facial electromyography (fEMG). In particular, reaction to an unpleasant stimulus is associated with an increase in fEMG activity in the *corrugator supercilii* muscle [51]. Facial EMG activity was collected using two electrodes placed in the brow region [52]. The electrodes were connected to a wireless preamplifier and the signal was sampled at 1,000Hz. The raw fEMG signals were processed using an in-house Matlab® program. A threshold-based procedure was used to obtain the physiologically significant phasic activity (i.e., stimulus-related activity): phasic activity was considered as physiologically significant when the processed fEMG signal was twice the standard deviation of the baseline reference. Afterwards, physiologically significant phasic fEMG responses were calculated by computing the Root Mean Square (RMS) value [53].

Self-reported behavioral intentions were evaluated using four questions which respondents answered when exposed to cigarette packs. These related to the following: desire to stop smoking, desire to reduce cigarette consumption, desire to seek information on quitting and the urge to smoke; these were accompanied by a five-point Likert scale (1: strongly disagree; 2: disagree; 3: moderately agree; 4: agree; 5: strongly agree).

### Participants and procedure

Fifty-three French daily smokers were recruited by a market research company and were asked their age, sex, occupation and number of cigarettes smoked per day. France was chosen to conduct this research because tobacco use is widespread– 34.6% of the French population are smokers [54]–and because all tobacco packs in France are plain and have displayed larger TVWs since January 1^st^ 2017 (the law came into effect on May 20^th^ 2016, after our research) [55]. A final total of 48 of the 53 recruited smokers participated in the study, which lasted approximately one hour. In the sample, 29 participants were female; participants’ profiles varied (they comprised students, workers, mid-level employees, etc.); the average age was 29 (with the youngest aged 19, and the oldest 57); and participants smoked an average of 9.02 cigarettes per day.

After the usual informed-consent procedure, participants answered a questionnaire on their personal characteristics (age, gender, activities, etc.) before being fitted with the EDA and fEMG signal collection device. Electrophysiological signals were collected with the Biopac MP150 Data Acquisition System, which was synchronized to the experimental procedure (stimuli exposure) using E-Prime research software. The 48 participants were presented with eight cigarette packs (participants all saw the eight selected pictures), representing the different experimental conditions: two threat levels (moderate/high); two image sizes (40/75%); and two pack types (plain/branded) (S1 Appendix). The order in which the stimuli appeared was counterbalanced for each participant by block randomization. The experimental procedure involved watching eight packs of cigarettes on a screen (with four highly and four moderately TVWs), each displayed successively for 4.5 seconds (within-subjects experiment). Respondents filled in the iconic SAM scale for each stimulus. After the measurement materials had been removed, participants were asked to look at the eight packs again and to fill in the negative emotion and behavioral intention self-report scales. At the end of the study, they were thanked and presented with a €15 gift card. The research design was approved by the Ethics Committee of the Graduate School of Management at the University of Rennes (France).

### Analysis

Statistical analysis has been performed for this within-subjects experiment using SPSS (v.20).

Factorial repeated measures ANOVA tests were conducted for all the indices considered after data normality and sphericity were verified. In order to explore the main effects of threat levels on emotional responses and behavioral intentions and, from an exploratory perspective, the multiplier effect of the size of images and the context in which they are displayed, two series of analyses were conducted: the main and interaction effects of (1) threat level and type of pack, and (2) threat level and image size. An interaction test between image size and type of pack was also performed.

## Results

### Main and interaction effects of threat level and type of pack

There was a significant main effect of the threat level on emotional responses (Table 1). Respondents were aroused when exposed to the TVWs. Self-reported emotional arousal was significantly higher for highly TVWs than for moderately TVWs, *F*(1,30) = 66.21, *p* = .000, *r* = .83. Physiological emotional arousal (electrodermal activity) was significantly higher for highly TVWs than for moderately TVWs, *F*(1,29) = 12.02, *p* = .002, *r* = .54. Respondents felt displeasure and negative emotions when exposed to the test TVWs. Self-reported pleasure was significantly lower for highly TVWs than for moderately TVWs, *F*(1,30) = 34.99, *p* = .000, *r* = .73. Fear and disgust were significantly greater for highly than for moderately TVWs, *F*(1,30) = 129.62, *p* = .000, *r* = .90. Physiological negative emotions (fEMG) increased significantly for both highly and moderately TVWs, showing that they both induced negative reactions (i.e., phasic activity was at least twice the standard deviation of the EMG baseline). fEMG responses to highly TVWs were greater from those induced by the moderately TVWs, but the difference was not significant, *F*(1,30) = 1.76, *p* = .194.

**Table 1:** Effects of threat level and pack type on emotional responses.

	Moderate threat	High threat	Threat effects	Branded pack	Plain pack	Type effects	Interaction Threat x Type
	Mean *(SD)*	Mean *(SD)*	*F* *(p value)* *effect size* *observed power*	Mean *(SD)*	Mean *(SD)*	*F* *(p value)* *effect size* *observed power*	*F* *(p value)* *effect size* *observed power*
**Emotional arousal**							
Self-reported arousal	2.66 *(*.*14)*	3.43 *(*.*13)*	**66.21 *(*.*000)* .*83 1***	3.03 *(*.*15)*	3.06 *(*.*12)*	.12 *(*.*726)* .*06* .*064*	.56 *(*.*459)* .*14* .*112*
Electrodermal activity	.145 *(*.*025)*	.226 *(*.*034)*	**12.02 *(*.*002)* .*54* .*918***	.179 *(*.*029)*	.193 *(*.*030)*	.35 *(*.*559)* .*11* .*088*	***4*.*59 (*.*041)* .*37* .*545***
**Emotional valence**							
Self-reported pleasure	1.93 *(*.*11)*	1.32 *(*.*07)*	**34.99 *(*.*000)* .*731***	1.64 *(*.*08)*	1.61 *(*.*08)*	.13 *(*.*724)* .*06* .*064*	1.68 *(*.*205)* .*23* .*241*
Self-reported fear and disgust	2.89 *(*.*13)*	4.04 *(*.*12)*	**129.62 *(*.*000)* .*90 1***	3.36 *(*.*12)*	3.56 *(*.*13)*	3.17 *(*.*085)* .*30* .*407*	1.01 *(*.*323)* .*18* .*163*
fEMG activity *(corrugator)*	.024 *(*.*004)*	.027 *(*.*004)*	1.76 *(*.*194)* .*23* .*251*	.026 *(*.*004)*	.026 *(*.*004)*	.05 *(*.*832)* .*04* .*055*	1.65 *(*.*208)* .*23* .*238*

In bold: significant results

The type of pack had no apparent significant isolated effect on emotional responses (Table 1).

Regarding the interaction effects of threat level and type of pack on emotional responses, a significant effect on physiological emotional arousal (electrodermal activity) was observed, *F*(1,29) = 4.59, *p* = .041, *r* = .37 (Table 1). For branded packs, increasing the threat level of the TVWs did not lead to any significant variation in the level of arousal: P2 (moderately TVWs and branded packs) = P4 (highly TVWs and branded packs). In contrast, increasing the threat level on plain packs led to significant variations in the level of arousal: P1 (moderately TVWs and plain packs) < P3 (highly TVWs and plain packs). Highly TVWs associated with a plain pack (P3) were therefore the most effective combination for provoking an intense emotional reaction (S3 Appendix).

Behavioral intentions were also altered by exposure to TVWs. There was a significant and main effect of the threat level on behavioral intentions (Table 2). The desire to stop smoking was significantly greater for highly TVWs than for moderately TVWs, *F*(1,30) = 37.85, *p* = .000, *r* = .75, as well as the desire to reduce cigarette consumption, *F*(1,30) = 31.46, *p* = .000, *r* = .72. In addition, the urge to smoke a cigarette after the experiment was significantly lower following exposure to highly TVWs than to moderately TVWs, *F*(1,30) = 13.26, *p* = .001, *r* = .55. Finally, the desire to find out information about quitting smoking was significantly higher following exposure to highly TVWs than to moderately TVWs, *F*(1,30) = 19.61, *p* = .000, *r* = .63.

**Table 2:** Effects of threat level and pack type on behavioral intentions.

	Moderate threat	High threat	Threat effects	Branded pack	Plain pack	Type effects	Interaction Threat x Type
	Mean *(SD)*	Mean *(SD)*	*F* *(p value)* *effect size* *observed power*	Mean *(SD)*	Mean *(SD)*	*F* *(p value)* *effect size* *observed power*	*F* *(p value)* *effect size* *observed power*
**Desire to stop smoking**	2.46 *(*.*16)*	3.07 *(*.*20)*	**37.85 *(*.*000)* .*75 1***	2.71 *(*.*18)*	2.82 *(*.*19)*	.88 *(*.*356)* .*17* .*148*	.40 *(*.*532)* .*11* .*094*
**Desire to reduce cigarette smoked**	2.74 *(*.*18)*	3.35 *(*.*19)*	**31.46 *(*.*000)* .*72 1***	2.98 *(*.*19)*	3.11 *(*.*19)*	.92 *(*.*344)* .*17* .*154*	.06 *(*.*807)* .*04* .*057*
**The urge to smoke**	2.10 *(*.*16)*	1.80 *(*.*15)*	**13.26 *(*.*001)* .*55* .*941***	2.03 *(*.*16)*	1.87 *(*.*15)*	**5.77 *(*.*023)* .*40* .*642***	.44 *(*.*511)* .*40* .*099*
**Desire to look for info**	1.91 *(*.*15)*	2.31 *(*.*18)*	**19.61 *(*.*000)* .*63* .*990***	2.09 *(*.*16)*	2.14 *(*.*17)*	.34 *(*.*565)* .*10* .*087*	.61 *(*.*442)* .*14* .*117*

In bold: significant results.

Pack type had a more limited effect on behavioral intentions: only one item–“urge to smoke”–was significantly lower for the plain pack condition than for the branded pack condition, *F*(1,30) = 5.77, *p* = .023, *r* = .40.

Finally, no interaction effect between threat level and pack type was observed.

### Main and interaction effects of threat level and image size

As mentioned before, there was a significant and main effect of the threat level on emotional responses (Table 3). In this test, fEMG responses to highly TVWs were significantly higher than those induced by the moderately TVWs, *F*(1,27) = 4.06, *p* = .054, *r* = .36. There was also a significant, and large, main effect of the threat level on behavioral intentions (Table 4). Image size had no apparent significant isolated effect on emotional responses or behavioral intentions (Tables 3 and 4). Nevertheless, two significant interaction effects of threat level and image size were observed on emotional responses (Table 3).

**Table 3:** Effects of threat level and image size on emotional responses.

	Moderate threat	High threat	Threat effects	Image 40%	Image 75%	Size effects	Interaction Threat x Size
	Mean *(SD)*	Mean *(SD)*	*F* *(p value)* *effect size* *observed power*	Mean *(SD)*	Mean *(SD)*	*F* *(p value)* *effect size* *observed power*	*F* *(p value)* *effect size* *observed power*
**Emotional arousal**							
Self-reported arousal	2.75 *(*.*15)*	3.38 *(*.*16)*	**24.91 *(*.*000)* .*70* .*998***	3.06 *(*.*14)*	3.06 *(*.*16)*	.00 *(1)* .*00* .*050*	.04 *(*.*838)* .*04* .*055*
Electrodermal activity	.149 *(*.*03)*	.254 *(*.*04)*	**13.44 *(*.*001)* .*60* .*939***	.205 *(*.*03)*	.197 *(*.*03)*	.24 *(*.*629)* .*10* .*076*	.33 *(*.*569)* .*11* .*086*
**Emotional valence**							
Self-reported pleasure	2.09 *(*.*12)*	1.36 *(*.*07)*	**64.04 *(*.*000)* .*84 1***	1.80 *(*.*11)*	1.66 *(*.*09)*	2.44 *(*.*130)* .*29* .*325*	2.06 *(*.*163)* .*27* .*282*
Self-reported fear and disgust	2.71 *(*.*16)*	4.07 *(*.*12)*	**114.51** ***(*.*000)*** **.*90*** ***1***	3.30 *(*.*16)*	3.49 *(*.*12)*	2.64 *(*.*116)* .*30* .*347*	**5.40 *(*.*028)* .*41* .*610***
fEMG activity *(corrugator)*	.016 *(*.*00)*	.020 *(*.*00)*	**4.06 *(*.*054)* .*36* .*493***	.016 *(*.*00)*	.019 *(*.*00)*	1.65 *(*.*210)* .*24* .*236*	**8.95 *(*.*006)* .*50* .*822***

In bold: significant results.

**Table 4:** Effects of threat level and image size on behavioral intentions.

	Moderate threat	High threat	Threat effects	Image 40%	Image 75%	Size effects	Interaction Threat x Size
	Mean *(SD)*	Mean *(SD)*	*F* *(p value)* *effect size* *observed power*	Mean *(SD)*	Mean *(SD)*	*F* *(p value)* *effect size* *observed power*	*F* *(p value)* *effect size* *observed power*
**Desire to stop smoking**	2.69 *(*.*19)*	3.18 *(*.*22)*	**16.40 *(*.*000)* .*62* .*974***	2.92 *(*.*22)*	2.96 *(*.*19)*	.09 *(*.*763)* .*05* .*060*	.05 *(*.*822)* .*04* .*055*
**Desire to reduce cigarette smoked**	2.90 *(*.*21)*	3.51 *(*.*20)*	**17.14 *(*.*000)* .*63* .*978***	3.22 *(*.*21)*	3.18 *(*.*19)*	.10 *(*.*754)* .*06* .*061*	.17 *(*.*687)* .*08* .*068*
**The urge to smoke**	2.18 *(*.*20)*	1.91 *(*.*19)*	**6.95 *(*.*014)* .*46* .*718***	2.06 *(*.*20)*	2.04 *(*.*19)*	.04 *(*.*830)* .*04* .*055*	.06 *(*.*805)* .*04* .*057*
**Desire to look for info**	2.15 *(*.*21)*	2.56 *(*.*22)*	**15.98 *(*.*000)* .*62* .*970***	2.34 *(*.*22)*	2.37 *(*.*20)*	.08 *(*.*776)* .*06* .*059*	1.97 *(*.*173)* .*26* .*272*

In bold: significant results.

First, a significant effect was observed on physiological negative emotions (fEMG), *F*(1,27) = 8.95, *p* = .006, *r* = .50 (Table 3). When image size was 40%, varying the threat level had no significant effect: P1 (moderately TVWs and size 40%) = P3 (highly TVWs and size 40%). Conversely, when the image size increased to 75%, increasing the threat level amplified negative reactions: P2 (moderately TVWs and size 75%) < P4 (highly TVWs and size 75%). Thus, a highly threatening and large image (P4) was the most effective combination for inducing the most negative emotional reactions (S4 Appendix).

Secondly, a significant interaction effect of threat level and image size on self-reported negative emotions (fear, disgust) was observed *F*(1,26) = 5.40, *p* = .028, *r* = .41 (Table 3). The strongest negative emotions were induced by the highly TVWs, regardless of size: P3 (highly TVWs and size 40%) = P4 (highly TVWs and size 75%). By contrast, large and moderately TVWs provoked stronger negative emotions: P2 (moderately TVWs and size 75%) > P1 (moderately TVWs and size 40%) (S5 Appendix).

### Interaction effect between image size and pack type

Finally, a significant interaction effect of image size and pack type was observed in the desire to reduce cigarette consumption *F*(1,46) = 5.40, *p* = .025, *r* = .32 (S6 Appendix). When an image covered 75% of the pack, no significant effect on desire to reduce tobacco consumption was observed for plain or branded packs: P1 (branded packs and size 75%) = P2 (plain packs and size 75%). On the contrary, when the image covered 40% of the pack, it was significantly more effective in provoking a desire to reduce cigarette consumption when displayed on a plain vs. a branded pack: P3 (branded packs and size 40%) < P4 (plain packs and size 40%). Thus, branded packs with a warning covering 40% of the pack were the least effective in making people want to reduce their tobacco consumption, and plain packs with a warning covering 40% were the most effective.

## Discussion

Combining different measurements enabled us to (i) better understand the persuasive process of TVWs, warning size, and pack type; (ii) identify reactions that cannot be verbalized by smokers and (iii) inform the debate on the effectiveness of TVWs. This was achieved by measuring emotions and behavioral intentions through self-reports and, for the first time, psychophysiological measurements. As emotional episodes precede smokers’ behavioral intentions [20–25], and are predictable drivers of decision-making [56], it was important to detect them.

A contribution of this study is to show that moderately and highly TVWs provoke emotional reactions among daily smokers. While, in the literature, conflicting results have been reported about the effects of TVWs and messages that appeal to fear (little or no effect vs. positive influence) [57], the methodology used in this study shows that smokers not only generate convenient emotional responses (“I gave the answer that was expected of me”) but also experience bodily emotional episodes involving a process of change in different subsystems (physiological reactions). The use of physiological measurements of emotions appears particularly relevant to establishing a more complete and exact measurement of the emotions felt.

The findings reveal that the level of threat affects smokers’ emotional reactions in different ways: the more threatening the TVWs, the stronger the resulting negative emotional reactions: a higher self-reported and physiological level of arousal, a significant decrease in self-reported pleasure, a stronger self-reported feeling of fear and disgust, and higher corrugator activity. By using a more accurate method to measure emotions, these results confirm that the threat level of warnings and its effect on negative emotions are key variables in explaining the impact of TVWs. All measured emotional reactions vary in the same way.

Type of pack and image size have no apparent significant isolated effect on emotional responses, but our study reveals interesting interactions between the threat level and type of pack, and the threat level and image size, which affect emotional responses. On the one hand, highly TVWs associated with a plain pack were the most effective combination for provoking a significant emotional reaction (S3 Appendix). Plain packs (compared with branded packs) increase the physiological activation in smokers when they are exposed to highly TVWs, but smokers did not seem to be sufficiently aware of this to express it (there is no difference on self-reported arousal reactions) [29]. On the other hand, highly TVWs associated with image sizes of 75% were the most effective combination for inducing the strongest negative emotional reactions (S4 and S5 Appendices).

The findings reveal that the level of threat also affects smokers’ behavioral intentions: the more threatening the TVWs, the stronger the desire to stop smoking, reduce tobacco consumption, and find information on quitting, and the lower the urge to smoke a cigarette. All measured behavioral intentions vary in the same way.

Pack type and image size have no apparent significant isolated effect on behavioral intentions, except for one measure. We note that the urge to smoke was significantly lower for the plain-pack condition than for the branded-pack condition. This study does not reveal interaction effects between the level of threat and the type of pack or the image size on behavioral intentions. However, an interaction effect between the image size and the type of pack was identified: for an image size of 40%, displaying warnings on plain packs (compared with branded packs) increased their impact on the desire to reduce cigarette consumption (S6 Appendix).

To sum up, our research reveals that enhancing the level of threat of warnings increased emotional reactions among smokers and, combining this with changing the image size (75%) and pack type (plain pack) is even more effective. The impact of the level of threat is also significant on behavioral intentions, but changing the image size and pack type did not increase its effect.

A number of reasons may explain this result. It is possible that one-off exposure to packs was not enough. It would be worthwhile conducting further research to determine if repeated exposure has an impact on responses. Cultural and contextual reasons may also provide an explanation since it has been shown that plain packs combined with highly TVWs have less of an impact on behavioral intentions (reducing cigarette cravings) among French adolescents than among US adolescents [37]. This can be explained by the fact that there is a higher frequency of smoking among French youths and, therefore, they may feel a greater level of negative reaction when exposed to prevention devices. As a consequence, the impact of plain packaging and warnings size may take more time to influence behaviors in countries like France. Finally, the profile of our sample (daily smokers) may also explain why effects were mainly observed on emotional reactions. According to theoretical models of the persuasive impact of threatening messages [58], it is advisable to juxtapose them with reassuring ones (e.g., the positive effects of giving up smoking, encouragement to quit, information on quitting, etc.) to increase their impact on behaviors [59, 60]. Recent research has underlined that the combination of TVWs with positive and salient information on/in packs (inserts, quit-line telephone numbers, etc.) could prompt quitting attempts in smokers and willingness to seek help. It can explain why our stimuli did not lead to behavioral change as they were presented without positive information.

Our research makes a number of contributions to questions of public health, but it also has some limitations. First, material constraints surrounding the use of psychophysiological methods required a relatively modest sample size of 48 smokers. However, the size of the effect and its observed power were satisfactory, and this was entirely within the standards of studies that use such methods (25–50 test participants in most studies). Second, the complexity of our experimental design, which included a large number of both independent and dependent variables, was not ideal and may have increased the potential for Type 1 errors. The exploratory approach regarding the multiplier effect of image size and the context in which it is used in this study requires further elaboration. The inconclusive results for these variables suggests a need to rethink their measures of efficiency. Moreover, intentions, not real behaviors, were measured in this study. Finally, because young non-smokers are an important target for public health actors, the same research should be conducted on adolescents to analyze the effects of TVWs on emotional reactions and intentions not to start smoking.

In spite of these limitations, our research is useful for public health stakeholders. It underlines the benefits of increasing the threat level of TVWs. Whereas more and more countries are using moderately and highly TVWs, our research suggests that the optimal level of threat seems to be the high level. For countries where text-only warnings, which provoke low negative emotional reactions, are still used (the United States, China, etc.), this option is probably not the best to increase the effectiveness of warnings. Increasing the size of images and their placement context must also be considered as they both increase the emotional impact of TVWs. Considering these findings, the recent decisions to adopt plain packaging and larger TVWs in different countries present a key opportunity for public health (in Australia, the UK, France, Ireland, Canada, New Zealand, Hungary, etc.) and are in line with the FCTC of the WHO [61].

## Supporting information

S1 Appendix Description of the eight packs/warnings that respondent No. 4 was exposed to. (DOCX)

S2 Appendix Scales used for valence and arousal self-report. (DOCX)

S3 Appendix Interaction effect between threat level and type of pack on physiological arousal. (DOCX)

S4 Appendix Interaction effect between threat level and image size on physiological negative emotions. (DOCX)

S5 Appendix Interaction effect between threat level and image size on self-reported negative emotions. (DOCX)

S6 Appendix Interaction effect between type of pack and image size on desire to reduce cigarette consumption. (DOCX)
