# Supplementary material for: The influence of threatening visual warnings on tobacco packaging: Measuring the impact of threat level, image size, and type of pack through psychophysiological and self-report methods
Source: PLoS One. 2017 Sep 14;12(9):e0184415. doi: 10.1371/journal.pone.0184415 (PMC5598963; doi:10.1371/journal.pone.0184415)
Supplement: S1 Appendix — (DOCX) [file pone.0184415.s001.docx]

**S1 Appendix. Description of the eight packs/warnings that Respondent No. 4 was exposed to**

|  | **Highly TVWs** | | **Moderately TVWs** | |
| --- | --- | --- | --- | --- |
| **Size**  **75%** | *Branded packs* | | *Plain packs* | |
|  | A photograph of a Marlboro pack that displayed a warning combining **a close-up** (1) **of a foot with gangrene** and the text (at the bottom): “Smoking clogs your arteries.” ^(a)^ | A photograph of a Marlboro pack that displayed a warning combining **a close-up** (2) **of a foot with gangrene** and the text (at the bottom): “Smoking clogs your arteries.” ^(a)^ | A photograph of a Camel pack that displayed a warning combining **a wide-angle shot** (1) **of the face of a man with a hole in the throat** and the text (at the bottom): “Smoking causes mouth and throat cancer.” ^(b)^ | A photograph of a Marlboro pack that displayed a warning combining **a wide-angle shot** (2) **of the face of a man with a hole in the throat** and the text (at the bottom): “Smoking causes mouth and throat cancer.” ^(b)^ |
| **Size**  **40%** | *Plain packs* | | *Branded packs* | |
|  | A photograph of a Camel pack that displayed a warning combining **a close-up** (1) **of a tongue with cancer** and the text (at the bottom): “Smoking causes mouth and throat cancer.” ^(b)^ | A photograph of a Marlboro pack that displayed a warning combining **a close-up** (2) **of a mouth with a large scar due to cancer** and the text (at the bottom): “Smoking causes mouth and throat cancer.” ^(b)^ | A photograph of a Camel pack that displayed a warning combining **a wide-angle shot** (1) **of a man who had lost a leg, due to gangrene, sitting in a wheelchair** and the text (at the bottom): “Smoking clogs your arteries.” ^(a)^ | A photograph of a Camel pack that displayed a warning combining **a wide-angle shot** (2) **of a man who had lost a leg, due to gangrene, sitting in a bed** and the text (at the bottom): “Smoking clogs your arteries.” ^(a)^ |

^(a)^ Similar warning pictures to the ones we used are available at: [www.tobaccolabels.ca/healthwarningsinfo/theme/?n=Health%20Effects%20%E2%80%93%20Vascular%20System](http://www.tobaccolabels.ca/healthwarningsinfo/theme/?n=Health%20Effects%20%E2%80%93%20Vascular%20System) or [www.who.int/tobacco/healthwarningsdatabase/healtheffects/en/](http://www.who.int/tobacco/healthwarningsdatabase/healtheffects/en/).

One of the pictures we used (a warning used in the European Union since 2016) is available at: <http://eur-lex.europa.eu/legal-content/FR/TXT/PDF/?uri=CELEX:32014L0109&from=EN> (“série 1”, page L 360/24)

^(b)^ Similar warning pictures to the ones we used are available at: [www.tobaccolabels.ca/healthwarningsinfo/theme/?n=Health%20Effects%20%E2%80%93%20Mouth](http://www.tobaccolabels.ca/healthwarningsinfo/theme/?n=Health%20Effects%20%E2%80%93%20Mouth) or [www.who.int/tobacco/healthwarningsdatabase/healtheffects/en/](http://www.who.int/tobacco/healthwarningsdatabase/healtheffects/en/).

One of the pictures we used (a warning used in Chile from 2006 to 2007) is available at <http://www.tobaccolabels.ca/countries/chile/>
