# Supplementary material for: The influence of threatening visual warnings on tobacco packaging: Measuring the impact of threat level, image size, and type of pack through psychophysiological and self-report methods
Source: PLoS One. 2017 Sep 14;12(9):e0184415. doi: 10.1371/journal.pone.0184415 (PMC5598963; doi:10.1371/journal.pone.0184415)

**S2 Appendix. Scales used for valence and arousal self-report**

Valence: Imagine that you are the character below. When I look at this pack of cigarettes, I feel a positive emotion (pleasure) or a negative emotion (displeasure).


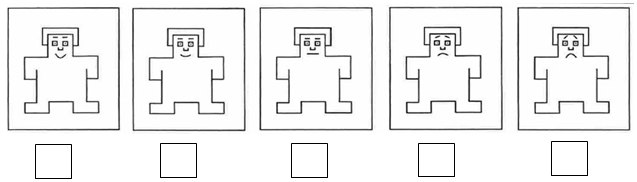


Arousal: Imagine that you are the character below. When I look at this pack of cigarettes, I feel tense and excited, or calm and relaxed.


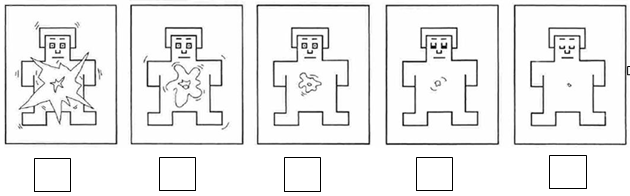

Supplement: S2 Appendix — (DOCX) [file pone.0184415.s002.docx]
