# Supplementary material for: The influence of threatening visual warnings on tobacco packaging: Measuring the impact of threat level, image size, and type of pack through psychophysiological and self-report methods
Source: PLoS One. 2017 Sep 14;12(9):e0184415. doi: 10.1371/journal.pone.0184415 (PMC5598963; doi:10.1371/journal.pone.0184415)

**S5 Appendix. Interaction effect between threat level and image size on self-reported negative emotions**


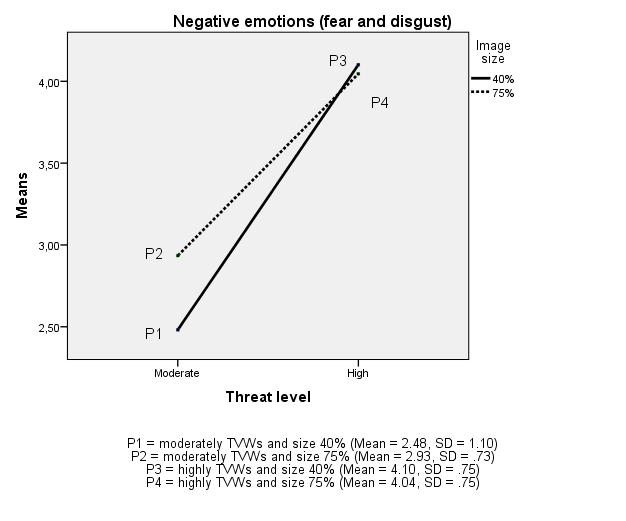

Supplement: S5 Appendix — (DOCX) [file pone.0184415.s005.docx]
